# The effect of active warming in prehospital trauma care during road and air ambulance transportation - a clinical randomized trial

**DOI:** 10.1186/1757-7241-19-59

**Published:** 2011-10-21

**Authors:** Peter Lundgren, Otto Henriksson, Peter Naredi, Ulf Björnstig

**Affiliations:** 1 Division of Surgery, Department of Surgery and Perioperative Sciences, Umeå University, Sweden

**Keywords:** hypothermia, body temperature regulation, thermal comfort, active warming, passive warming, prehospital trauma care, emergency medical services (EMS)

## Abstract

**Background:**

Prevention and treatment of hypothermia by active warming in prehospital trauma care is recommended but scientifical evidence of its effectiveness in a clinical setting is scarce. The objective of this study was to evaluate the effect of additional active warming during road or air ambulance transportation of trauma patients.

**Methods:**

Patients were assigned to either passive warming with blankets or passive warming with blankets with the addition of an active warming intervention using a large chemical heat pad applied to the upper torso. Ear canal temperature, subjective sensation of cold discomfort and vital signs were monitored.

**Results:**

Mean core temperatures increased from 35.1°C (95% CI; 34.7-35.5°C) to 36.0°C (95% CI; 35.7-36.3°C) (p < 0.05) in patients assigned to passive warming only (n = 22) and from 35.6°C (95% CI; 35.2-36.0°C) to 36.4°C (95% CI; 36.1-36.7°C) (p < 0.05) in patients assigned to additional active warming (n = 26) with no significant differences between the groups. Cold discomfort decreased in 2/3 of patients assigned to passive warming only and in all patients assigned to additional active warming, the difference in cold discomfort change being statistically significant (p < 0.05). Patients assigned to additional active warming also presented a statistically significant decrease in heart rate and respiratory frequency (p < 0.05).

**Conclusions:**

In mildly hypothermic trauma patients, with preserved shivering capacity, adequate passive warming is an effective treatment to establish a slow rewarming rate and to reduce cold discomfort during prehospital transportation. However, the addition of active warming using a chemical heat pad applied to the torso will significantly improve thermal comfort even further and might also reduce the cold induced stress response.

**Trial Registration:**

ClinicalTrials.gov: NCT01400152

## Background

In a cold, wet or windy environment, an injured or ill person is often exposed to a considerable cold stress. Heat loss is often aggravated due to exhaustion, light, torn or wet clothing, major bleeding, entrapment or the administration of cold intravenous fluids or sedative drugs and admission hypothermia is an independent risk factor associated with worse outcome and higher mortality in trauma patients [1-6]. The cold induced stress response will also render great thermal discomfort which might increase the experience of pain and anxiety, even in still normothermic patients [7]. Thus, in addition to immediate care for life threatening conditions, actions to reduce cold exposure and prevent further heat loss is an important and integrated part of prehospital primary care. Initial measures should be taken to get the patient into shelter, remove wet clothing and insulate the patient from ambient weather conditions and ground chill within adequate wind- and waterproof insulation ensembles (passive warming). In addition, depending on the victim's physiological status, body core temperature, available resources and expected duration of evacuation, the application of external heat (active warming) is in most guidelines recommended to be considered to aid in protection from further cooling during evacuation and transport to definitive care [8-12].

Several studies on mildly hypothermic (body core temperature, T_co _= 32-35°C) shivering subjects have found that exogenous skin heating attenuates shivering heat production by an amount equivalent to the heat donated [13-15]. Thus, in a mildly hypothermic shivering victim, external warming generally does not decrease afterdrop or increase rewarming rate, however it might provide other advantages including increased comfort, decreased cardiac work and preserved substrate availability. When shivering is diminished or absent, as in moderate (T_co _= 28-32°C) to severe (T_co _< 28°C) hypothermia or otherwise impaired due to the overall medical condition of the patient (i.e. old age, alcohol or drug ingestion, head or spinal injury, severe trauma or depleted metabolic energy substrates) some form of exogenous external or internal heat is required, otherwise afterdrop will continue and little or no rewarming will occur [16,17].

Accordingly, effective prehospital field treatment of patients exposed to cold stress is considered of utmost importance to improve the medical condition on admission to the emergency room and active warming already in the field is considered one important part of such treatment. Since the warming modalities need to be portable and easily handled by Search and Rescue (SAR) or Emergency Medical Services (EMS) personnel there are limited treatment options in the field or during transport to definitive care. Chemical heat pads, hot water bottles, plumbed water filled blankets, charcoal fueled heat pacs, forced air warming and resistive heating devices are commonly used and advised [8-12], but the lack of studies in field conditions is noticed [18] and to the authors' knowledge, only two randomized clinical trials have evaluated the effectiveness of such modalities in the field [19,20].

We therefore decided to evaluate the effect of an active warming intervention on cold stressed trauma patients using chemical heat pads, previously evaluated in a laboratory study [17], as one possible field applicable warming device during road or air ambulance transportation of trauma patients. Primary outcome measures were body core temperature, cold discomfort and vital signs.

## Methods

### Design and settings

The study was designed as a randomized, clinical trial of prehospital active warming intervention for trauma patients, where enrolled patients were assigned to either passive warming with blankets (routine care) or passive warming with blankets with the addition of an active warming intervention using a large chemical heat pad applied to the upper torso. Ethical approval was obtained from the Regional Ethical Review Board at Umeå University. The study was conducted from December 2007 until May 2010. Fourteen road ambulance units and one helicopter unit, serving a primarily suburban area in the northern parts of Sweden with about 125 000 inhabitants, were selected for the study. After given both written and verbal instructions, the participating EMS personnel carried out the study as a part of their normal duty, without interference by the investigators.

### Population

Subjects were sequential trauma patients, age ≥ 18 years, who had sustained an injury outdoors and were transported by one of the participating EMS units. Patients were excluded if initial level of consciousness was affected, (Glasgow Coma Scale < 15), or if duration of transportation was expected to be shorter than 10 minutes. As the aim of the study was to investigate the effect of active warming intervention in cold stressed patients, those patients who had already received active warming or had been taken indoors for more than 10 minutes before EMS unit arrival or had an initial cold discomfort rating ≤ 2 were also excluded.

### Protocol

At the scene of injury event, all patients initially received routine trauma care, including passive warming with blankets. After loading into the ambulance or helicopter, informed consent to be part of the study was obtained. Enrolled patients then were selected for either passive warming or passive warming with the addition of active warming by opening of sequentially numbered and sealed envelopes containing randomized study protocols. A tympanic sensor was placed in the patient's ear canal and the outer ear sealed with a soft insulation cover. After 5 minutes an initial recording of ear canal temperature, cold discomfort, heart rate, blood pressure and respiratory rate, was obtained before active warming was begun if assigned. Apart from air temperature set to 25°C in the transportation unit, no other regulations were appointed. The number of blankets applied and specific care, such as immobilization or intravenous fluids and medications were provided according to standard trauma protocols. Repeated recordings of ear canal temperature, cold discomfort and vital signs were obtained every 30 minutes and upon arrival to the receiving hospital or health care center.

### Passive warming

The participating ambulance units all had polyester blankets (200 × 135 × 0.4 cm, 1.200 g, 2.4 clo), woollen blankets (190 × 135 × 0.5 cm, 1.900 g, 2.7 clo) and one rescue blanket (nylon outer with synthetic filling and cotton inner, 275 × 125 × 0.7 cm, 2.300 g, 3.6 clo) as part of their standard equipment. The type and number of blankets applied in each case were selected according to the EMS crew judgement without any regulations by the investigators. For comparative reasons the polyester blanket was accounted for as 1.0 blanket whereas the woollen blanket was accounted for as 1.1 blankets and the rescue blanket was accounted for as 1.5 blankets depending on their thermal insulation value (clo) determined according to European Standard for assessing requirements of sleeping bags [21].

### Active warming intervention

A chemical heat pad (Dorcas AB, Skattkärr, Sweden), was selected as the active warming device. In a previous laboratory study this chemical heat pad, applied both to the anterior and posterior upper torso, was appreciated for its effectiveness in transferring heat to a cold person [17]. To simplify for the EMS crew, in this study the chemical heat pad on the posterior upper torso was left out. After activation, the heat pad (42 × 25 × 2 cm), reaching about 50°C within 2 minutes, was applied across the anterior upper torso, leaving only one layer of thin clothing between the heat pad and the skin. If the clothing had to be removed to gain necessary access to the patient, the heat pad was placed in an ordinary pillow-case to prevent burns to the skin. Following the initial chemical reaction, the surface temperature of the heat pad gradually declines [17]. To maintain effective heat transfer during longer transportations, the heat pad was thus replaced every 30 minutes.

### Monitoring

A closed ear canal temperature sensor (Smiths Medical, Ltd., UK) was selected to monitor core temperature changes (± 0.2°C) during transportation. Ear canal temperature has been shown to correlate well with oesophageal temperature [22,23]. If properly sealed from the ambient air, closed ear canal temperature is also reliable in subzero and wind conditions [22] and thus considered the most accurate non invasive method of measuring body core temperature in the field [10-12]. After visual inspection of the outer ear to rule out any injuries, the sensor was gently placed in the middle of the ear canal. In addition to the outer soft cell foam cylinder that conforms to the ear canal and seals out ambient air, a soft insulation cover was placed on the outer ear and secured with Velcro around the head. The ear canal sensor was then connected to a temperature monitor (Novamed, Inc., USA) and left in place during the whole transportation.

Cold discomfort was monitored using a numerical rating scale [24], whereby the subjects estimated their sensation of cold to the whole body, not specific body parts, providing values from 0 to 10, where 0 indicated no sensation of cold and 10 indicated unbearable sensation of cold.

Vital signs were monitored using routine equipment and data collection sheets were filled out during transportation by the EMS personnel. In addition to ear canal temperature, vital signs, cold discomfort and overall satisfaction of care, the following information was recorded: time from injury to EMS unit arrival, on-scene duration, transportation time, outdoor temperature, wind speed, ambulance unit indoor temperature, patient characteristics, clothing characteristics, the type and number of blankets applied, immobilization and the administration of warm intravenous fluids and medications.

### Data analysis

According to pre-study power calculations, with an estimated difference in core temperature of ≥ 0.5°C or cold discomfort rating of ≥ 2, an alpha of 0.05 and a power of 0.90, the minimum number of patients required to achieve statistical significance was 21 in each group and the study was ended after, with some margin, a sufficient number of patients had successfully been enrolled. Groups were compared using Mann-Whitney U-test for interval and ordinal data and Chi-2 or Fisher's exact test for nominal data, whereas pair wise related variable comparisons was made using the Wilcoxon Signed-Rank test. In addition, change in cold discomfort rating was characterized as increased, unchanged or decreased and the difference between groups was analyzed using Fisher's exact test. Statistical significance was defined as p < 0.05.

## Results

### Patient characteristics

Fifty-one trauma patients were enrolled in the study. Of these, one patient wished to end the study prior to arrival to the receiving hospital and two were excluded because of breach of protocol (assigned intervention was not given). Thus, a total of 48 patients, all subjected to blunt trauma, with a mean coded Revised Trauma Score (RTS) [25] of 7.83 (range 7.55 - 7.84), successfully completed the study, being randomized to either passive warming with blankets (n = 22) or passive warming with blankets with the addition of active warming (n = 26). The included patients were 19 male and 29 female and there were no significant differences between the two groups on morphometric or demographic characteristics (table 1).

**Table 1:** Patient characteristics and confounding factors

	Passive warming (n = 22)	Active warming (n = 26)
		
**Patient characteristics**		
Gender (male/female)	9/13	10/16
Age (years)	45 (34 - 55)	43 (36 - 50)
Body Mass Index	25.0 (22.8 - 27.3)	25.4 (23.6 - 27.3)
**Environment**		
Outdoor temperature (°C)	-6 (-9 - -2)	-3 (-6 - -1)
Outdoor wind speed (m/s)	2 (1 - 3)	2 (1 - 4)
Interior unit temperature (°C)	20 (19 - 21)	20 (19 - 21)
Cold exposure (min)	64 (41 - 88)	81 (61 - 101)
Time to 2^nd ^measurement (min)	24 (21 - 28)	27 (24 - 29)
Total transportation (min)	33 (25 - 41)	37 (25 - 49)
Clothing (light/medium/heavy)	5/6/9	4/9/13
Clothing (dry/moist/wet)	13/2/4	21/3/1
**Treatment during transport**		
No. of blankets	2.7 (2.2 - 3.2)	2.3 (1.9 - 2.7)
Undress (none/partial/total)	12/8/2	14/10/1
Whole body fixation (yes/no)	8/13	8/17
Intravenous fluids (ml)	91 (31 - 151)	50 (0 - 107)
Intravenous opioids (yes/no)	10/12	14/12
Intravenous sedatives (yes/no)	2/20	5/21

Values are mean (95% confidence interval) or number of patients. The internal drop-out of any variable was ≤ 3 patients and there are no significant differences between groups (p < 0.05).

### Environment

The average ambient air temperature at the scene of accident was -4 ± 7°C (mean ± SD) and the average time from the injury until the patient was loaded into the EMS unit (cold exposure) was 73 ± 53 minutes with no significant differences between the two groups. The mean interior unit temperature during transport was 20 ± 3°C and the mean number of blankets applied was 2.5 ± 1.1 with no significant differences between the two groups. There were also no significant differences between the two groups in distribution of clothing thickness or wetness, the extent of undressing, the incidence of whole body fixation, the amount of intravenous fluids transfused or the incidence of intravenous opioids or sedatives administered during transport (table 1).

### Primary outcome

The average transportation time to the receiving hospital or health care centre was 35 ± 26 minutes (mean ± SD) with no significant differences between the two groups. Thus, at the second measurement, performed at an average of 26 ± 7 minutes all 48 subjects were included, whereas at the third measurement, performed at an average of 58 ± 5 minutes only 12 subjects remained. The analysis of primary outcome variables was therefore terminated after the second measurement.

Mean initial ear canal temperature was 35.1°C (95% CI; 34.7 - 35.5°C) in patients assigned to passive warming only and 35.6°C (95% CI; 35.2 - 36.0°C) in those assigned to additional active warming with no significant differences between the two groups. At the second measurement, mean ear canal temperatures in both groups were significantly increased to 36.0°C (95% CI; 35.7 - 36.3°C) and 36.4°C (95% CI; 36.1 - 36.7°C) respectively with no significant differences between the two groups (table 2).

**Table 2:** Primary outcome

	Passive warming (n = 22)	Active warming (n = 26)
**Body core temperature **(°C) *		
1^st ^measurement	35.1 (34.7 - 35.5)	35.6 (35.2 - 36.0)
2^nd ^measurement	36.0 (35.7 - 36.3) †	36.4 (36.1 - 36.7) †
Cold discomfort **		
1^st ^measurement	5 (4 - 7)	7 (5 - 8)
2^nd ^measurement	3 (0 - 5) †	2 (1 - 3) †
↳ increased	1	0
↳ unchanged	5	0
↳ decreased	15	26 ‡
Vital signs *		
Heart rate		
1^st ^measurement	83 (77 - 90)	84 (78 - 90)
2^nd ^measurement	82 (76 - 87)	80 (75 - 86) †
Systolic blood pressure		
1^st ^measurement	138 (129 - 147)	136 (127 - 145)
2^nd ^measurement	134 (124 - 143)	131 (124 - 139)
Respiratory rate		
1^st ^measurement	17 (16 - 19)	18 (16 - 20)
2^nd ^measurement	17 (15 - 18)	16 (14 - 18) †
Revised Trauma Score	7.84 (7.84 - 7.84)	7.83 (7.80 - 7.84)

Values are * mean (95% confidence interval) or ** median (interquartile range) and number of patients. The internal drop-out of any variable was ≤ 2 patients.

† Significant difference within the same group (Mann-Whitney U-test, p < 0.05)

‡ Significant difference between groups (Fisher's exact test, p < 0.05)

The initial median cold discomfort rating in patients assigned to passive warming only was 5 (IQR; 4 - 7) and the initial median cold discomfort rating in patients assigned to passive warming with the addition of active warming was 7 (IQR; 5-8) with no significant differences between the two groups. At the second measurement, cold discomfort was significantly reduced in both groups. However, in the group assigned to passive warming only, 15 out of 22 patients presented a decrease in cold discomfort, whereas in the group assigned to additional active warming all 26 patients presented a decrease in cold discomfort ratings, the difference in cold discomfort change being statistically significant (table 2).

There were no statistically significant differences in initial vital signs between the two groups. At the second measurement, the vital signs were statistically unchanged for the patients assigned to passive warming only, whereas patients assigned additional active warming presented a small but statistically significant reduction in mean heart rate and respiratory frequency (table 2).

## Discussion

### Overview

This study evaluates the effectiveness of active warming in prehospital trauma care using a large chemical heat pad applied to the upper torso in addition to passive warming with blankets during transportation to definitive care. Over the first 30 minutes of prehospital transportation, both patients receiving passive warming only and patients receiving passive warming with the addition of active warming presented a statistically significant increase in body core temperature as well as improved cold discomfort. However, in the group assigned to passive warming only, 2/3 of the patients presented a decrease in cold discomfort, whereas all patients in the group assigned to additional active warming presented a decrease in cold discomfort ratings, the difference in cold discomfort change being statistically significant.

### Possible mechanism for findings

In previous laboratory studies on mildly hypothermic shivering subjects, exogenous skin heating has been shown to attenuate shivering heat production by an amount equivalent to the heat donated [13-15]. Accordingly, in this study, enrolling trauma patients with an initial body core temperature of about 35°C and preserved shivering capacity, active warming had no additional effect on body core temperature compared to passive warming only. In contrast, two previous randomized clinical trials found a decrease in body core temperature with passive warming only, whereas with additional active warming using either electrically heated blankets [19] or multiple chemical heat pads [20], body core temperature was increased during transportation. Since passive warming only as an adequate treatment alternative presupposes intact shivering capacity and enough insulation in relation to cold stress and ambient environmental conditions, differences regarding these factors might explain differences between studies.

Although body core temperature was increased, only 2/3 of the patients assigned to passive warming only presented a decrease in cold discomfort whereas all patients assigned to additional active warming presented a decrease in cold discomfort during transportation. This beneficial effect on thermal comfort by application of a chemical heat pad to the upper torso is probably explained by a combination of reduction in shivering thermogenesis and increased skin temperature. Although shivering was not monitored per se in this study, a reduction of the cold induced stress response was indicated by a small but statistically significant decrease in respiratory frequency and heart rate in patients assigned to active warming, whereas patients assigned to passive warming presented no significant change in these parameters during transportation.

### Practical implications

Admission hypothermia is an independent risk factor associated with worse outcome in trauma patients and previous retrospective analysis of trauma registries as well as prospective clinical studies have reported significant changes in physiologic variables, such as increased oxygen consumption, depletion of energy stores, disruption of blood clotting mechanisms, increased fluid resuscitation requirements, immune suppression and development of organ failure already at mild hypothermic states compared to normothermic trauma patients [1-6].

Owing to peripheral vasoconstriction, the temperature in the periphery of the body starts to decline long before body core temperature is affected. After removal from the cold environment there is a temperature equalisation between the warm body core and the cold peripheral parts contributing to a continuous fall in body core temperature, designated the afterdrop phenomenon. The magnitude of the afterdrop, which can be considerable and amount to several degrees, is dependent on temperature gradients in the tissues, peripheral circulation and endogenous heat production. Thus, initial measures in prehospital care of cold stressed patients are aiming at avoiding further heat loss to the environment and reducing the amount and duration of the afterdrop [8-12].

According to this study on cold stressed trauma patients with an initial body core temperature of about 35°C and preserved shivering capacity, passive warming, if adequate, is an effective treatment to prevent afterdrop, establish a steady rewarming rate and reduce cold discomfort during transportation to definitive care. However, additional active warming had a beneficial effect in improving thermal comfort and indicated a small reduction of the cold induced stress response. Even in these mild hypothermic states, active warming might be of considerable clinical importance, especially in scenarios with diminished to absent shivering or inadequate passive warming. In a sustained cold outdoor environment, such as in prolonged extrications or in multiple casualty situations where available insulation often is inadequate, shivering will then be maintained in order to prevent afterdrop, thereby increasing respiratory and circulatory demands which might be detrimental for an already compromised patient. The application of external heat would therefore be even more important to reduce shivering strain. Also, if shivering is diminished or absent due to moderate or severe hypothermia or due to the patient's overall medical condition some form of exogenous heat is most likely required, otherwise afterdrop will continue and little or no rewarming will occur [16,17]. Improved thermal comfort might also relieve the experience of pain and anxiety and contribute to the physiological well-being of the patient during prehospital care.

### Limitations

In addition to body core temperature, subjective sensation of cold discomfort and vital signs, other parameters such as oxygen consumption (as a measure of shivering) and skin temperature would have been important and useful supplements as indicators of cold stress.

### Further research

The thermal effectiveness of active warming in prehospital trauma care has only been evaluated in a few previous clinical trials [19,20] and the results are diverging. Various degrees of injuries as well as different warming modalities and different amounts of passive warming might explain differences between the studies. All studies are also relatively small and included patients suffering from not more than mild hypothermia. Thus, thermal effectiveness of active warming in prehospital trauma care deserves further research, especially including more severely injured patients suffering from moderate or severe hypothermia.

### Conclusion

In mildly hypothermic trauma patients, with preserved shivering capacity, adequate passive warming is an effective treatment to establish a slow rewarming rate and to reduce cold discomfort during prehospital transportation. However, the addition of active warming using a chemical heat pad applied to the torso will significantly improve thermal comfort even further and might also reduce the cold induced stress response.

## Competing interests

The authors declare that they have no competing interests.

## Authors' contributions

The authors contibuted in the following way to the paper:

PL: Design of the study, aquisition of data, analysis and interpretation of data and writing of the manuscript.

OH: Design of the study, aquisition of data, analysis and interpretation of data and writing of the manuscript.

PN: Interpretation of data and critically revising the manuscript.

UB: Design of the study, interpretation of data and critically revising the manuscript.

All authors read and approved the final manuscript.

## References

[B1] Ireland S Endacott R Cameron P Fitzgerald M Paul E The incidence and significance of accidental hypothermia in major trauma - A prospective observational study Resuscitation 2011 82 3 300 306 10.1016/j.resuscitation.2010.10.016 21074927

[B2] Beilman GJ Blondet JJ Nelson TR Nathens AB Moore FA Rhee P Puyana JC Moore EE Cohn SM Early hypothermia in severly injured trauma patients is a significant risk factor for multiple organ dysfunction syndrome but not mortality Ann Surg 2009 249 5 845 50 10.1097/SLA.0b013e3181a41f6f 19387315

[B3] Martin RS Kilgo PD Miller PR Hoth JJ Meredith JW Chang MC Injury associated hypothermia: an analysis of the 2004 National Trauma Data Bank Shock 2005 24 2 114 8 10.1097/01.shk.0000169726.25189.b1 16044080

[B4] Wang HE Callaway CW Peitzman AB Tisherman SA Admission hypothermia and outcome after major trauma Critical Care Medicine 2005 33 6 1296 301 10.1097/01.CCM.0000165965.31895.80 15942347

[B5] Shafi S Elliott AC Gentilello L Is hypothermia simply a marker of shock and injury severity or an independent risk factor for mortality in trauma patients? Analysis of a large national trauma registry Journal of Trauma-Injury Infection & Critical Care 2005 59 5 1081 5 16385283 10.1097/01.ta.0000188647.03665.fd

[B6] Gentilello LM Jurkovich GJ Stark MS Hassantash SA O'Keefe GE Is hypothermia in the victim of major trauma protective or harmful? A randomized, prospective study Annals of Surgery 1997 226 4 439 47 discussion 447-9 10.1097/00000658-199710000-00005 9351712 PMC1191057

[B7] Robinson S Benton G Warmed blankets: an intervention to promote comfort to elderly hospitalized patients Geriatric nursing 2002 23 320 323 10.1067/mgn.2002.130273 12494005

[B8] Danzl DF Auerbach P Accidental Hypothermia Wilderness medicine 2007 5 Philadelphia. PA: Mosby Elsevier 125 159

[B9] Giesbrecht GG editor Hypothermia frostbite and other cold injuries prevention, survival, rescue and treatment 2006 2 Seattle, WA: Mountaineers Books

[B10] Socialstyrelsen Hypothermia: cold injuries and cold water near drowning 2002 2 Stockholm, Sverige: The National Board of Health and Welfare (Socialstyrelsen)

[B11] Durrer B Brugger H Syme D Elsensohn F The medical on site treatment of hypothermia Consensus Guidelines on Mountain Emergency Medicine and Risk Reduction 2001 1 Italy: Casa editrice stefanoni - lecco 71 75

[B12] State of Alaska cold injuries guidelines Alaska Emergency Medical Services Program, Department of Health and Social Services, Juneau http://www.chems.alaska.gov/EMS/documents/AKColdInj2005.pdf Accessed 19 April 2011

[B13] Giesbrecht GG Bristow GK Uin A Ready AE Jones RA Effectiveness of three field treatments for induced mild (33.0°c) hypothermia J Appl Physiol 1987 63 2375 79 3436871 10.1152/jappl.1987.63.6.2375

[B14] Sterba JA Efficacy and safety of prehospital rewarming techniques to treat accidental hypothermia Ann Emerg Med 1991 20 896 901 10.1016/S0196-0644(05)81434-9 1854075

[B15] Giesbrecht GG Sessler DI Mekjavic IB Schroeder M Bristow GK Treatment of mild immersion hypothermia by direct body-to-body contact J Appl Physiol 1994 76 2373 9 7928860 10.1152/jappl.1994.76.6.2373

[B16] Giesbrecht GG Goheen MS Johnston CE Kenny GP Bristow GK Hayward JS Inhibition of shivering increases core temperature afterdrop and attenuates rewarming in hypothermic humans J Appl Physiol 1997 83 1630 4 9375331 10.1152/jappl.1997.83.5.1630

[B17] Lundgren JP Henriksson O Pretorius T Cahill F Bristow G Chochinov A Pretorius A Bjornstig U Giesbrecht GG Field Torso Warming Modalities: A Comparative Study Using a Human Model Prehosp Emerg Care 2009 3 371 378 10.1080/10903120902935348 19499476

[B18] Allen PB Salyer SW Dubick MA Holcomb JB Blackbourne LH Preventing hypothermia: comparison of current devices used by the US Army in an in vitro warmed fluid model J Trauma 2010 69 Suppl 1 S154 61 20622611 10.1097/TA.0b013e3181e45ba5

[B19] Kober A Effectiveness of resistive heating compared with passive warming in treating hypothermia associated with minor trauma: a randomized trial Mayo Clin Proc 2001 76 369 375 10.4065/76.4.369 11322352

[B20] Watts DD The utility of traditional prehospital interventions in maintaining thermostasis Prehosp Emerg Care 1999 3 115 122 10.1080/10903129908958918 10225643

[B21] EN 13537 Requirements for sleeping bags 2002 Brussels: European Committee for Standardization

[B22] Walpoth BH Galdikas J Leupi F Muehlemann W Schlaepfer P Althaus U Assesment of hypothermia with a new "tympanic" thermometer J Clin Monit 1994 10 91 96 10.1007/BF02886820 8207458

[B23] Webb GE Comparison of esophageal and tympanic temperature monitoring during cardiopulmonary bypass Anesth Analg 1973 52 5 729 33 4542341

[B24] Parsons KC Parsons KC Thermal comfort Human thermal environments: the effects of hot, moderate, and cold environments on human health, comfort and performance 2003 2 London, UK: Taylor & Francis 196 228

[B25] Champion HR Sacco WJ Copes WS Gann DS Gennarelli TA Flanagan ME A revision of the trauma score J Trauma 1989 29 5 623 9 10.1097/00005373-198905000-00017 2657085

